# Prevalence of *Chlamydia trachomatis* infection in parturient women in Gipuzkoa, Northern Spain

**DOI:** 10.1186/s40064-016-2268-4

**Published:** 2016-05-10

**Authors:** Luis Piñeiro, Arantza Lekuona, Gustavo Cilla, Izaskun Lasa, Laura-Pilar Martinez-Gallardo, Javier Korta, Emilio Pérez-Trallero

**Affiliations:** Microbiology Department, Hospital Universitario Donostia, Paseo Dr Beguiristain s/n, 20014 San Sebastián, Spain; Obstetrics and Gynecology Department, Hospital Universitario Donostia, San Sebastián, Spain; Biomedical Research Centre Network for Respiratory Diseases (CIBERES), San Sebastián, Spain; Pediatrics Department, Hospital Universitario Donostia, San Sebastián, Spain; Department of Medicine Preventive and Public Health, UPV/EHU, San Sebastián, Spain

**Keywords:** *Chlamydia trachomatis*, Prevalence, Pregnant women, Parturient women, Asymptomatic women, Spain

## Abstract

The prevalence of *Chlamydia trachomatis* infection in Southern Europe is poorly understood and its identification is essential for the design of appropriate prevention policies. The prevalence of *C. trachomatis* in 2011–2014 was determined through polymerase chain reaction in urine samples from 11,687 unselected parturient women from the Basque Country, Spain (San Sebastián area). The overall age-adjusted prevalence was 1.0 % (95 % CI 0.8–1.2). The prevalence of infection in women younger than 25 years was 6.4 % and decreased substantially with increasing age: 2.0 % in 25–29 year-olds and 0.5 % in older women (*P* < 0.001). The prevalence was higher in parturient of foreign origin (1.9 %, 95 % CI 1.3–2.5) than in Spanish parturients (0.8 %, 95 % CI 0.6–1.0), (*P* < 0.001). The results of this study support the need to screen young women as part of antenatal care in Spain.

## Background

*Chlamydia trachomatis* (*C. trachomatis*) is the most diagnosed sexually transmitted bacterial infection worldwide, with an estimated 100 million new infections each year (WHO 2012). Chlamydial infections in women frequently cause cervicitis and urethritis, but it has been estimated that up to 70–75 % of the cases are asymptomatic. Asymptomatic infection facilitates its spread to sexual contacts and can cause serious sequelae, including pelvic inflammatory disease, tubal damage, ectopic pregnancy, and infertility (Cates and Wasserheit 1991; Haggerty et al. 2010). In pregnant women, the infection has been associated with adverse pregnancy outcomes such as prematurity, and may be transmitted vertically to the newborn, which may result in neonatal ocular or respiratory tract infection (Blas et al. 2007; Rours et al. 2011; Hammerschlag 2011).

The knowledge of the prevalence of the *C. trachomatis* infection is essential for the design of appropriate infection control programs. In most countries of Southern Europe, the prevalence of *C. trachomatis* infection in pregnant women is not well characterized. Moreover, even though these women are a potential target group for prevention policies (ECDC 2014; CDC et al. 2014; Böhm et al. 2009), to date no recommendation has been made in Spain in this regard. The aim of this study was to estimate the prevalence of *C. trachomatis* infection in an unselected population of pregnant women at term, by age-group and geographical area of origin, in the Basque Country, Spain.

## Methods

The study was conducted in the city of San Sebastián and surrounding counties (413,000 inhabitants, with 4000–4500 deliveries per year). Eighty-seven percent of parturient women in the area attended Donostia University Hospital, the remainder attended private health care facilities.

The presence of *C. trachomatis* was determined in urine samples (first catch) collected from unselected parturient women who delivered a live neonate in the Donostia University Hospital between January 2011 and December 2014. *C. trachomatis* determination was included in a previously established free-of-charge prevention program for sexually-transmitted diseases in our hospital, which included serological detection of *Treponema pallidum* and human immunodeficiency virus (HIV). Women were informed about *C. trachomatis* infections and were screened in the immediate postpartum period, unless they refused to participate. Samples were preserved at 4 °C until processing, which was carried out within 48 h. *C. trachomatis* DNA was detected using a real-time PCR (Cobas^®^ 4800 CT/NG, Roche Diagnostics, Branchburg, NJ, USA), which also detects *Neisseria gonorrhoeae* DNA. Samples with an invalid result (no amplification of the internal control) were diluted appropriately and were re-analyzed. Detection of antibodies against *Treponema pallidum* and HIV was performed with a chemiluminescent microparticle immunoassay (CMIA) (Architect Syphilis TP reagent, and Architect HIV Ag/Ab Combo, Abbott Laboratories, Wiesbaden, Germany). *Treponema* CMIA-positive samples were reanalyzed using fluorescent treponemal antibody absorption (FTA-Abs) (Bio-Mèrieux, Marcy l´Ètoile, France) and Venereal Disease Research Laboratory (VDRL) (Siemens, Marburg, Germany) tests. Upon detection of *C. trachomatis* infection, women were referred to a gynecologist for treatment (azithromycin 1 g orally), as well as treatment of their sexual partners, if applicable. Similarly, their newborns were placed under medical and microbiological surveillance (medical examination and pharyngeal exudate 7–10 days after birth).

The prevalence of infection and its 95 % exact binomial confidence interval (CI) were calculated based on the number of *Chlamydia*-positive women (numerator) and on the number of women with a valid result of the diagnostic test (denominator). To estimate the prevalence according to the women’s geographical origin (native or foreign immigrant), the country of origin in all *Chlamydia*-positive women and in a sample of 1124 parturient women of the study was identified. As the proportion of immigrants vary widely with age, this sample was chosen by stratified random sampling in the four age-groups in which was divided the population of the study (sample size estimated with 5 % precision and 95 % confidence interval for each age-group) using as reference population that of parturient women from the Basque Country classified by age and country of origin, provided by the Basque Institute of Statistics. The distribution by countries found in the 1124 women was extrapolated to the population of women investigated. The overall prevalences were age-adjusted in relation to the distribution by the age of the total number of parturients in the area (Basque Institute of Statistics). The Chi square test was used to compare percentages with application of Fisher’s corrections (two-tailed), when required. A *P* value of <0.05 was considered statistically significant. The study was approved by the Ethics Committee for Clinical Research of the Health Area of Gipuzkoa.

## Results

Samples were analysed from 12,228 parturient women (median age 33 years, range 14–54), representing 76.4 % of the eligible population screened (84.6 % in the last three years). The result was invalid (inhibition of internal control amplification) in 541/12,228 samples (4.4 %), corresponding mostly to haematuric urine samples. *C. trachomatis* was detected in 121/11,687 women, with an overall age-standardized prevalence of 1 % (95 % CI 0.8–1.2) and an annual prevalence ranging from 1.2 % in 2011 to 0.8 % in 2013 (*P* = 0.14, non-significant). The median age of *C. trachomatis*–positive women was 27 years, range 17–43.

The prevalence of infection was higher in younger women, being 6.4 % in those aged <25 years, and decreased with increasing age: 2.0 % in 25–29 year-olds and 0.5 % in women aged ≥30 years old (*P* < 0.001) (Table 1). Women aged <25 and <30 years old represented 5.1 and 20.5 % of parturient women but accounted for 31.4 and 60.3 % of detected cases, respectively. Overall and in all age groups, prevalence was higher in immigrant than in Spanish parturient women [overall age-standardized prevalence 1.9 % (95 % CI 1.3–2.5) vs. 0.8 % (95 % CI 0.6–1.0) respectively, *P* < 0.001] (Table 1). Parturient women of Latin American origin had the highest prevalence (4.9 %) and they also were the main group of population among immigrants (43 %), accounting for 37.2 % of all infected women and 78.9 % (n = 45) of infected immigrants. The estimated prevalence in women of North African origin, the second largest immigrant group in Spain, was 1 % in this study (4/418).

**Table 1:** Prevalence of *C. trachomatis* infection by age and country of origin in puerperal women from the San Sebastián area, Basque Country, Spain (2011–2014)

Age^a^ (years)	Total				Native				Foreign immigrants				*P*
	No.	CT+	(%)	(95 % CI)	No.^b^	CT+	(%)	(95 % CI)	No.^b^	CT+	(%)	(95 % CI)	
<25	596	38	(6.4)^c^	(4.7–8.6)	247	14	(5.7)	(3.4–9.3)	349	24	(6.9)	(4.7–10.0)	0.552
25–29	1799	35	(2.0)	(1.4–2.7)	1164	16	(1.4)	(0.9–2.2)	635	19	(3.0)	(1.9–4.6)	0.018
30–34	4710	26	(0.6)	(0.4–0.8)	4065	18	(0.4)	(0.3–0.7)	645	8	(1.2)	(0.6–2.4)	0.019^d^
≥35	4582	22	(0.5)	(0.3–0.7)	4073	16	(0.4)	(0.2–0.6)	509	6	(1.2)	(0.6–2.5)	0.029^d^
Total	11,687	121	(1.0)^e^	(0.9–1.2)	9549	64	(0.7) ^e^	(0.5–0.8)	2138	57	(2.7)^e^	(2.1–3.4)	<0.001

^a^Median age 33 years, range 14–54

^b^Number estimated on the basis of randomized stratified sampling in women giving birth in the Donostia University Hospital during the study period (see “Methods”). The origin of *Chlamydia*-positive women was individually collected after their detection in screening

^c^Prevalence was 9.6 % (95 % CI 5.6–16.0) (12/125) and 5.5 % (95 % CI 3.8–8.0) (26/471) in women younger than 20 years and those aged 20–24 years old, respectively

^d^Fisher exact test (two tailed)

^e^Age-adjusted prevalence was 1.0 % (95 % CI 0.8–1.2) for the entire group and was 0.8 % (95 % CI 0.6–1.0) and 1.9 % (95 % CI 1.3–2.5) for native women and foreign immigrants, respectively, Chi square = 20.54, *P* < 0.001

Neither *Neisseria gonorrhoeae* nor current *Treponema pallidum* infection (VDRL positive) was detected in any of the 11,687 parturient women, although 27 women (0.2 %) (one of whom was *Chlamydia*-infected) had a history of syphilis (CMIA/FTA-Abs positive). Seven women (0.1 %) had HIV infection but none of these was coinfected with *C. trachomatis*. Only two *Chlamydia*-positive patients had symptoms attributable to the infection during pregnancy (one woman with cervicitis at week 25 and another with dysuria, leukocyturia and negative urine culture in week 36). A further four women had nonspecific symptoms of doubtful interpretation (vulvovaginal pruritus and/or vaginal discharge). The prevalence of preterm birth (neonates less than 37 weeks of gestational age) in *Chlamydia*-positive mothers was 7.6 % (n = 9). Perinatal transmission was detected in 10/91 (11 %) neonates studied. Four of them were symptomatic and presented conjunctivitis.

## Discussion

The present study was conducted in the immediate postpartum of women representative of the full-term pregnant women in our region that attended public healthcare. The median age at delivery in the San Sebastián area was high, which may explain why the overall prevalence of infection was considerably lower than in pregnant women from central and northern European countries (1 vs. 2–3.9 %) (Rours et al. 2011; Böhm et al. 2009; McMillan et al. 2006; Oakeshott et al. 2002; Kirk et al. 2008; Peuchant et al. 2015). As in most studies, the prevalence of infection in this study was higher in younger women. The prevalence of infection among women younger than 25 years (6.4 %) was closer to that found in the previously-mentioned European studies, showing figures somewhat higher than in Germany (4.5–5.7 %) (Böhm et al. 2009) but somewhat lower than in France (7.9 %) (Peuchant et al. 2015), the United Kingdom (8.6–8.7 %) (Oakeshott et al. 2002; Kirk et al. 2008), Eire (8.7 %) (McMillan et al. 2006) and the Netherlands (9.1 %) (Rours et al. 2011). The prevalence decreased sharply in older parturient women participating in this survey, which could partly be because women tend to have fewer partners as they get older. No *N. gonorrhoeae* infection was found in this large group, similar to the results recently obtained in the South of France in a group of 1004 pregnant women (Peuchant et al. 2015) and in Northern Italy among 2099 sexually active adolescents (Matteelli et al. 2016).

Information on the prevalence of *C. trachomatis* infection in the sexually-active general population in Spain is very scarce, since only one population-based study, limited to a town in the region of Asturias, has been conducted in the last decade (Fernández-Benítez et al. 2013). In that study, the prevalence of infection among sexually-active women younger than 25 years (4 %, range 2.8–6.4 %) was fairly similar to that observed in the present study. The prevalence of infection was higher in immigrant women as a whole, and especially in women of Latin American origin, who represent the main source of migration to Spain. These findings were probably related to the higher infection rates in their countries of origin (Cabeza et al. 2015; Pinto et al. 2011). Migration to Spain is a relatively recent event and, in studies conducted in other populations in Spain, has been considered an independent risk factor for *C. trachomatis* infection (Corbeto et al. 2010).

The study has some limitations. Because of the large number of pregnant women included in this study, no data on sexual activity were collected prospectively. Moreover, using nucleic acid amplification tests, the sensitivity of first catch urine specimens to detect *C. trachomatis* might be somewhat lower (up to 10 %) than that obtained from the vagina or cervix (CDC et al. 2014). Therefore the prevalence of *C. trachomatis* in women in Gipuzkoa could be slightly higher than that found in this study. However, first catch urine is an acceptable sample for the diagnosis of *C. trachomatis* urogenital infections in women (CDC et al. 2014), it is commonly used for *C. trachomatis* screening during pregnancy (Satterwhite et al. 2012; Böhm et al. 2009) and has been used in prevalence studies (Rours et al. 2011; McMillan et al. 2006). In addition, although lower percentages of invalid results have been referred by other authors (Rockett et al. 2010) they did not test urine specimens from postpartum women, among which haematuric urines are common. The percentage of invalid tests in our study (4.4 %) was similar to that found in another study in parturient women from Brazil (Pinto et al. 2011) in which 3.7 % of women were excluded because of bleeding during sample collection. Finally, although women were warned that urine collection should be first catch, we cannot ensure that it was the collected sample in all cases.

The most effective measure to control *C. trachomatis* infection is population-based screening in adolescents and young adults, but it is difficult to implement (ECDC 2014). An additional policy is screening in pregnancy. This form of screening is easy to implement and can achieve high coverage, since pregnant women usually make use of prenatal care and are usually willing to undergo diagnostic tests that could prevent adverse pregnancy outcomes. Pregnancy-based screening would detect infected women who are therefore at risk of pregnancy complications, and of transmitting the infection to their infants during delivery (Rours et al. 2011; Hammerschlag 2011; ECDC 2014; CDC et al. 2010). Most of these women are asymptomatic and their infection would remain undetected without screening. In this context, the high prevalence observed in young women in this study supports the need to recommend *C. trachomatis* screening in pregnant women ≤25 years during their first gestational control visit in this region of Spain and the possible broadening of this strategy to women aged 25–29 years.

## Conclusion

The results of this large study show that prevalence of *C. trachomatis* infection was high among pregnant women younger than 25 years (6 %). *C. trachomatis* infection represents a significant public health problem in this Southern European region. A gestational screening should be implemented in young women as part of antenatal care in Spain.

## Abbreviations

*C. trachomatis*: *Chlamydia trachomatis*
CI: confidence interval
HIV: human immunodeficiency virus
PCR: polymerase chain reaction
CMIA: chemiluminescent microparticle immunoassay
FTA-Abs: fluorescent treponemal antibody absorption
VDRL: Venereal Disease Research Laboratory
ECDC: European Centre for Disease Prevention and Control
CDC: Centers for Disease Control and Prevention (USA)
